# Supplementary material for: Analyses and Correlation of Pathologic and Ocular Cutaneous Changes in Murine Graft versus Host Disease
Source: Int J Mol Sci. 2021 Dec 24;23(1):184. doi: 10.3390/ijms23010184 (PMC8745722; doi:10.3390/ijms23010184)
Supplement: Supplementary file 1 [file ijms-23-00184-s001.zip › ijms-1443946-supplementary.pdf]

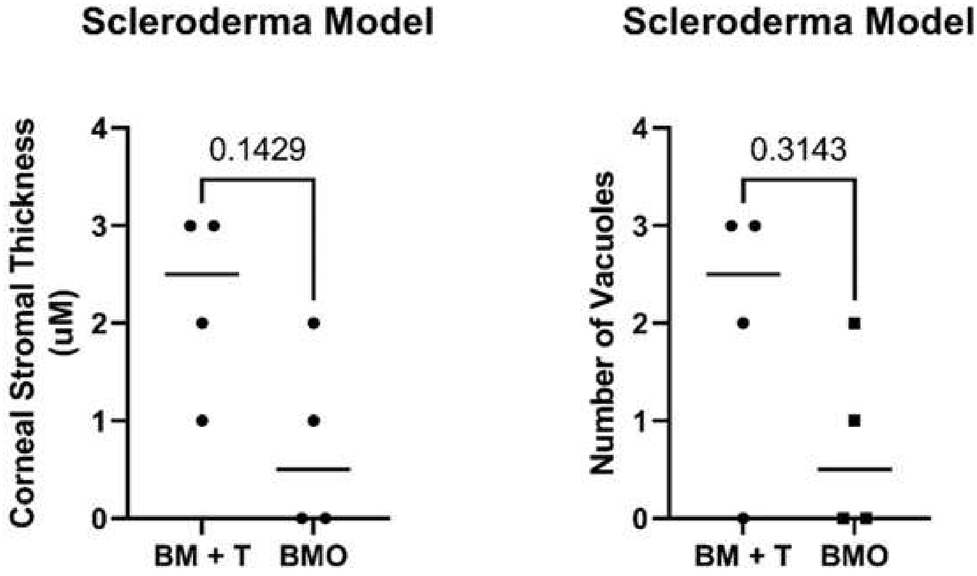

Figure S1: Histological analysis of cornea in the scleroderma model. Transplants were performed using donor B10.D2 (H2d) bone marrow + / - spleen cells containing T cells into conditioned BALB/c (H2d) recipients. BM+ T cells (n=4) and BMO (n=4). Corneal stromal thickness **(Left panel**) and number of vacuoles (**Right panel**) showed a trend (not statistically significant) to thickening and increased numbers in recipients of donor marrow + T cells.

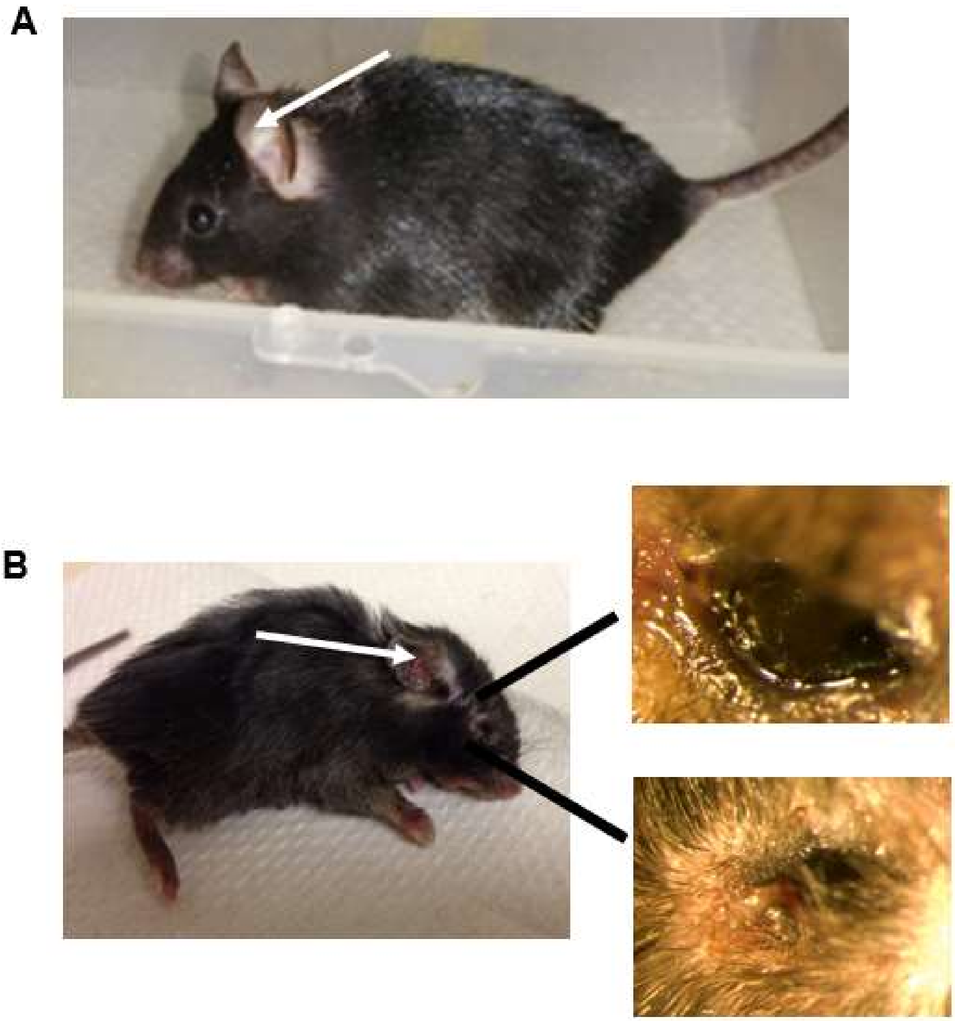

Figure S2: Development of ocular and skin changes following allogeneic HSCT between MHC-matched donors and recipients. A transplant was performed following TBI conditioning with donor LP/J (H2b) BM + / - spleen cells containing T cells into B6 (H2b) recipients. Representative mouse (n=4/gr). Examination of animals 5 weeks post-HSCT revealed ruffled fur, alopecia and scabbing in the ear (arrows) and paw skin. In the ocular compartment, edema with eyelid swelling was apparent in recipient of TCD-BM + T cells (**B**) but not TCD-BM alone (**A**).
